# Practical Implications of Policy Guidelines: A GIS Model of the Deployment of Community Health Volunteers in Madagascar

**DOI:** 10.9745/GHSP-D-19-00421

**Published:** 2020-09-30

**Authors:** Aurélie Brunie, James MacCarthy, Brian Mulligan, Yvette Ribaira, Andry Rabemanantsoa, Louisette Rahantanirina, Caleb Parker, Emily Keyes

**Affiliations:** a Health Services Research, FHI 360, Washington, DC, USA.; b Behavioral, Epidemiological and Clinical Sciences, FHI 360, Durham, NC, USA.; c Community Capacity for Health Program, JSI Research & Training Institute, Inc., Antananarivo, Madagascar.; d Reproductive, Maternal, Newborn and Child Health, FHI 360, Durham, NC, USA.

## Abstract

**Background:**

With increasing interest in strengthening community health programs nationally comes a need for operationalizing them in a realistic and achievable way. Limited information is available to help program managers establish appropriate parameters for their context. We examined aspects of program implementation related to deployment patterns of community health workers, called agents communautaires or ACs, in 2 districts of Madagascar.

**Methods:**

By analyzing program data and publicly available datasets in a geographic information system (GIS), we estimated the population and surface area coverage expected of ACs in 445 fokontany (communities). Additional modeling on travel time demands examined 1-way pedestrian travel time for ACs to receive routine support from their assigned health facilities and from socially marketed supply points under dry season conditions, as well as the impact on travel time based on ACs being reassigned to other facilities or supply points.

**Results:**

With the current distribution, ACs in 90% of fokontany have a catchment population of 1,000 or fewer people (2020 estimates) and ACs in 84% of fokontany have a catchment area of 25 km^2^ or less. We estimated that ACs in 58% of fokontany were located more than 2 hours from their supporting health facility, and the proportion of fokontany with ACs more than 2 hours away from their assigned supply point was 61%. Reassigning ACs to the closest facility or supply point led to modest improvements in those figures (7 and 4 percentage points, respectively).

**Conclusion:**

Findings allow visualizing the practical implications of coverage ratios for ACs to assess whether current demands are realistic. The physical access between ACs and the health system warrants significant attention due to challenges in transport and logistics. Analyses are timely to inform the Ministry of Public Health’s strategic thinking in the context of the development of the National Strategic Plan on Strengthening Community Health.

## Abstract (precis)

Geographic information systems can be used to support informed decisions about practical issues related to implementing community health worker (CHW) programs. Demands placed on CHWs regarding expected population and surface area coverage and travel time to facilities need to be carefully considered to ensure they are rational and realistic.

## Teaser

**Key Findings:**

- Community health workers (CHWs) in 23% of communities are assigned to cover either more than 1,000 people or more than 25 km^2^.
- In 58% of communities, CHWs were more than 2 hours away one way from the health facility; in 61% of communities, CHWs were more than 2 hours from their assigned supply point.

**Key Implications:**

- Program managers should consider assigning CHWs to the nearest health facility and placing supply facilities closer to decrease travel time.
- Policy makers should give latitude to communities and health centers to select and manage a number of volunteers matching local geographic and population characteristics.

## INTRODUCTION

Community health workers (CHWs) have been a vital component of primary health care since their inception close to 50 years ago.^1^ Although there are some examples of well-run CHW programs at scale in countries such as Brazil, Bangladesh, or Nepal,^2^^,^^3^ challenges have also been documented in scaling up and maintaining CHW programs, including poor planning, lack of coordination among actors, donor-driven management, siloed training, poor supervision and support, tenuous linkages with the health system, and lack of recognition of CHWs’ contributions.^4^^–^^6^ Recently, there has been renewed interest in expanding the use of CHWs to address shortages and misdistribution of health workers and to enable progress towards universal health coverage.^1^^,^^4^ This current drive and the accompanying commitment to integrate CHWs into health systems provide an opportunity to strengthen the design and performance of CHW programs.^1^^,^^4^^,^^5^

In 2018, the World Health Organization released evidence-based guidance on optimal health policy and system support to optimize the performance and impact of CHWs.^1^ In practice, however, CHW programs vary substantially and there is no global blueprint to maximize outputs and outcomes.^6^ For example, some CHWs are paid, others are volunteers, and some programs are specialized, but others are more extensive in the number of tasks CHWs are expected to perform. In addition, geography, the distance from a health facility, and cultural diversity provide additional layers of uncertainty and contextual variability. Program managers must find the right combination of key program elements to fit their context, including training, equipment, supplies, supervision, transport, financing, information systems, quality assurance and improvement, demand generation, governance, and incentives.^7^ One challenge that decision makers often face in ensuring plans and expectations are rational and realistic is a lack of adequate information on the existing health infrastructure, population, and geographic area. Some emerging tools are becoming available to support managers in operationalizing context-specific considerations for CHW programs, including the CHW coverage and capacity (C3) tool or the Community Health Planning and Costing Tool.^7^^,^^8^ To date, however, few resources are available to support program planning and management.

We used a geographic information system (GIS) to generate evidence on the reality of CHW program implementation in Madagascar to help program managers get a solid understanding of potential practical issues related to current deployment patterns for CHWs and support planning and management decisions. The published literature contains few examples of applying GIS to inform management of CHW programs. For example, a previous study used spatial data analysis and GIS methods to assess the role of geographical factors in variation of CHW performance, but another used GIS as a tool in calculating a cost prediction for implementing a CHW program in a specific area.^9^^,^^10^

We conducted 2 sets of analyses that are relevant in the context of Madagascar and suited to the use of GIS. The first set of analyses pertains to how many CHWs to deploy relative to population size and to the size of their catchment area. Establishing a realistic target population size typically depends on a constellation of factors including expected workload, frequency of contact required, services provided, expected time commitment, and local geography.^1^ Currently, some variability exists across countries in how coverage ratios are specified, with assignments for example being defined as a number of people or households per facility or per village.^11^ There is not always a clear understanding of how different metrics relate to each other or how they translate in terms of the geographic area CHWs may need to move around depending on their assigned tasks.

The second set of analyses examines travel time demands associated with maintaining functional linkages to the health system. In settings where CHWs are required to travel to health facilities for supervision and logistical support and supplies, they can incur additional transport and opportunity costs.^12^^–^^18^ Although reducing the costs for the populations CHWs serve is recognized as a significant advantage of CHW programs, costs borne by the CHWs are not always adequately considered as part of program design, planning, and implementation.^13^ A reality check to verify that assignment patterns of CHWs to health facilities are rational and travel time demands realistic based on health facility location, distance, terrain, and road system quality is important to ensure that programs are well-functioning and sustainable.

A reality check to verify that assignment patterns of CHWs are rational and travel time demands realistic is important for well-functioning, sustainable programs.

## METHODS

### Setting

The analyses examined the realities that volunteer CHWs face in performing their tasks in 2 districts of Madagascar (Mandritsara in Sofia region and Mananara Nord in Analanjirofo region) supported by the United States Agency for International Development (USAID) Community Capacity for Health Program, locally known as Mahefa Miaraka, implemented by JSI Research & Training Institute, Inc. Conducting this activity in Madagascar is timely because, after updating its National Community Health Policy in 2017, the Ministry of Public Health (MSANP) is currently developing the associated strategic plan.^19^^,^^20^ The main cadre of CHWs in Madagascar consists of volunteers called agents communautaires (ACs). Available estimates place the number of trained ACs at over 34,000 across the country.^21^ Order 8014-2009 stipulates a coverage ratio of 1 AC per village. In practice, implementing partners, in collaboration with the MSANP, support 2 ACs per fokontany (the lowest administrative level, equivalent to a collection of villages). Although the specific training and scope of practice of ACs tend to vary based on local needs and priority areas of support for implementing partners, ACs in the Mahefa Miaraka intervention area conduct health promotional activities; provide diagnosis and treatment for pneumonia, malaria, and diarrhea in under-5 children, as well as nutritional screening; and offer short-acting family planning methods, including condoms, pills, and injectables. Fokontany in the 2 districts are typically remote. ACs routinely conduct home visits and receive clients at the community health hut, called a toby. ACs receive technical supervision and malaria commodities from health centers called centres de santé de base (CSB), where they travel monthly to participate in review meetings and submit activity reports. Family planning and other child health products are obtained through an alternative socially marketed scheme operating through a network of supply points called points d’approvisionnement (PA).

### GIS Analysis Approach

Using multiple data sources and modeling within a GIS, we conducted 2 sets of analyses. In the first set, we examined the population and geographic coverage expected of ACs, as defined by the population-to-AC ratio and the surface-area-to-AC ratio. In the second set, we modeled the 1-way pedestrian travel time from the fokontany that the ACs serve to their assigned CSB under dry season conditions, as well as the 1-way pedestrian travel time to ACs’ assigned PA. Finally, we modeled the impact on travel time of scenarios whereby ACs were reassigned from their current CSB or PA to their closest CSB or PA. We used R version 3.5.1 for cleaning and plotting data, and ArcGIS Pro version 2.4.0 for all spatial analyses and map creation.^22^^,^^23^ This activity was reviewed by FHI 360’s Office of International Research Ethics and deemed to be exempt from ethical approval because it was not human subjects research.

We examined the population and geographic coverage expected of ACs, as well as their travel time by foot from their community to their assigned CSB.

### Expected Coverage Analysis

Through the MSANP, we obtained a database with the full list of ACs in the 2 districts, their fokontany and their assigned CSB, along with the geocoordinates of the CSB. We downloaded administrative boundaries for regions, districts, commune (administrative collection of fokontany), and fokontany in Madagascar from the United Nations Office for the Coordination of Humanitarian Affairs data portal, and manually matched fokontany names from the MSANP list to administrative boundary shape files.^24^

We estimated fokontany population by aggregating 2020 estimates of population per 100 by 100-m grid-cell produced by WorldPop.^25^ Fokontany surface areas were derived within the GIS using standard tools. Using information on the number of ACs assigned to each fokontany from the MSANP database, we calculated the population-to-AC and the surface-area-to-AC ratios for each fokontany. Where there were multiple ACs within the same fokontany, we assumed that population and surface area were split evenly among them.

To gain insight into the relationship between population and surface area coverage that each AC was responsible for, we calculated the proportion of fokontany where ACs were assigned both a population and a surface area below specific thresholds. Thresholds were chosen to represent common coverages based on the review of population-to-AC and surface-area-to-AC ratios for the 2 districts and were set at 1,000 people and 25 km^2^ respectively.

### Travel Time Analysis

#### Mapping of CSBs, PAs, and ACs

The location of each of the 56 CSBs was determined using CSB geocoordinates from the MSANP database and confirmed using high-resolution satellite imagery. We relocated 6 CSBs (11% of all CSBs). This included 2 CSBs with apparently incorrect locations (e.g., in dense forest or more than 2 km away from a building) that we relocated to the most densely populated area of the settlement in the fokontany of the same name through a visual assessment of the WorldPop data. An additional 4 CSBs that were missing geocoordinates in the database were added by georeferencing hand-drawn maps provided by Mahefa Miaraka field teams against current administrative boundaries and satellite imagery, then similarly locating the CSB in the most densely populated area of the largest nearby settlement through a visual assessment of the WorldPop data.

We used a list of PAs provided by Mahefa Miaraka and information on nearby landmarks to manually map the location of each PA. Where the exact location of the PA could not be ascertained, we centered the PA in the most populated area of the administrative commune they were in. We considered this assumption to be reasonable since PAs are typically co-located with small businesses and found in urban and peri-urban areas. Fourteen fokontany were excluded from analyses on travel time to PA because there was no PA identified for the corresponding commune.

Because we did not have information about where each AC was located, we approximated the location for ACs based on potentially habitable land in each fokontany using spatially disaggregated population data from WorldPop. We considered any area with 10 people or more per square kilometer to be habitable and a potential AC location. We chose a conservative population density threshold that contained more than 93% of the population across the 2 districts to ensure that we considered travel time requirements for ACs that live in urban and rural locations.

#### Cost Distance Raster

Since opportunities for mechanized travel are limited in the 2 districts, we used a cost distance raster (i.e., a grid of cells containing values reflecting the amount of time it takes to travel across each cell) to model travel time across roads and all terrain. We assumed travel by foot, as is typical for ACs in the 2 districts. To create the cost distance raster, each 100 by 100-m square cell was assigned a value representing the “cost” of traveling across the cell, expressed as a total time in minutes. The travel time cost for each cell was calculated by combining several layers of data to represent local conditions and assigning varied travel speeds corresponding to these conditions.

Local conditions were represented by the availability and types of roads, elevation, land cover, and rivers. The road network dataset was downloaded from OpenStreetMap after digitizing 885 km of roads and paths that were missing from the database for the 2 districts.^26^ The source of the geographic elevation was the 30-m resolution SRTM Digital Elevation Model (DEM), downloaded from the United States Geological Survey EarthExplorer portal, and land use data at 300-m resolution came from the European Space Agency’s GlobCover project.^27^^,^^28^

We used a baseline walking speed of 5 km/h for bare areas and all road types and reduced it for other land cover types (e.g., dense forests) to represent reduced walking speeds as in the GlobCover data.^29^^,^^30^ We used elevation data to model the effect of slope on travel speeds using Tobler’s hiking function and to generate a river network using the D8 flow accumulation method.^17^^,^^31^ We then used the Strahler stream order method to approximate the width of each river and defined river crossing speeds based on estimated widths and possible delays from waiting for a dugout canoe for medium rivers (20–60 m wide) or ferry for large rivers (over 60 m wide).^32^ Estimates of walking speed and river crossing delays were validated with Mahefa Miaraka field teams.

#### Travel Time

We used the cost distance raster to model 1-way fokontany-level pedestrian travel times by averaging travel times between potential AC locations within each fokontany and the CSB or PA assigned to the ACs from that fokontany. We then similarly modeled fokontany-level travel times to the closest CSB or PA. CSBs that did not have any ACs assigned to them in the MSANP database were not considered suitable for reassignment of ACs. All estimates represent travel times under dry season conditions and assume that each habitable cell in the raster has an equal probability of containing an AC.

## RESULTS

### Expected Coverage

**FIGURE 1.**
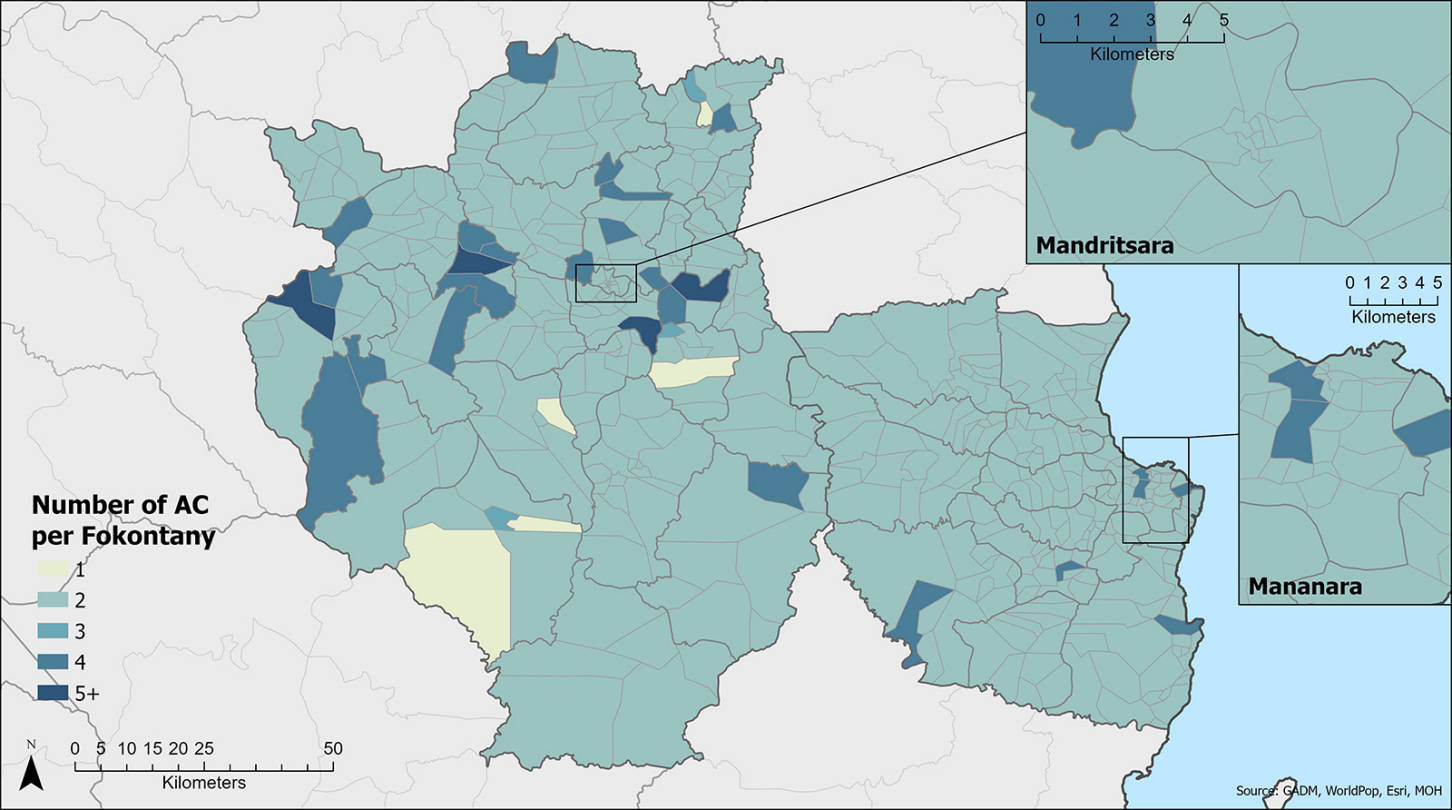
Number of ACs per Fokontany in Madagascar Abbreviation: ACs, agents communautaires (community health workers).

Table 1 shows characteristics of the 2 districts. Overall, 962 ACs were deployed across 445 different fokontany and supported by a network of 56 CSBs and 47 PAs. Three other CSBs did not support any ACs. The number of ACs per fokontany ranged from 1 to 6 (Figure 1); 92% of fokontany had 2 ACs, 7% had between 3 and 6, and the remaining 5 fokontany had fewer than 2. Imple-menting partners support the selection of 2 volunteers per fokontany as a matter of course. Additional volunteers can be added at the discretion of the CSB. Fokontany with more than 2 ACs most likely respond to greater coverage needs that arise from a larger population or surface area, a more dispersed population, geographic barriers, and/or different population subgroups (e.g., ethnic groups). Fokontany with less than 2 ACs likely are explained by 1 AC having recently stepped down and not yet been replaced, or where few people meet selection criteria.

**TABLE 1.** Characteristics of 2 Districts in Madagascar and Distribution of Community Health Workers

**District**	**Mandritsara**	**Mananara Nord**
Region	Sofia	Analanjirofo
Population	323,242	216,281
Number of fokontany	239	206
Number of CSB with ACs	35	19
Number of PA	30	17
Number of AC	530	432

Abbreviations: AC, agents communautaires (community health workers); CSB, centres de santé de base (health centers); PA, points d’approvisionnement (supply points).

Overall, 962 ACs were deployed across 445 different fokontany and supported by a network of 56 CSBs and 47 PAs.

**FIGURE 2.**
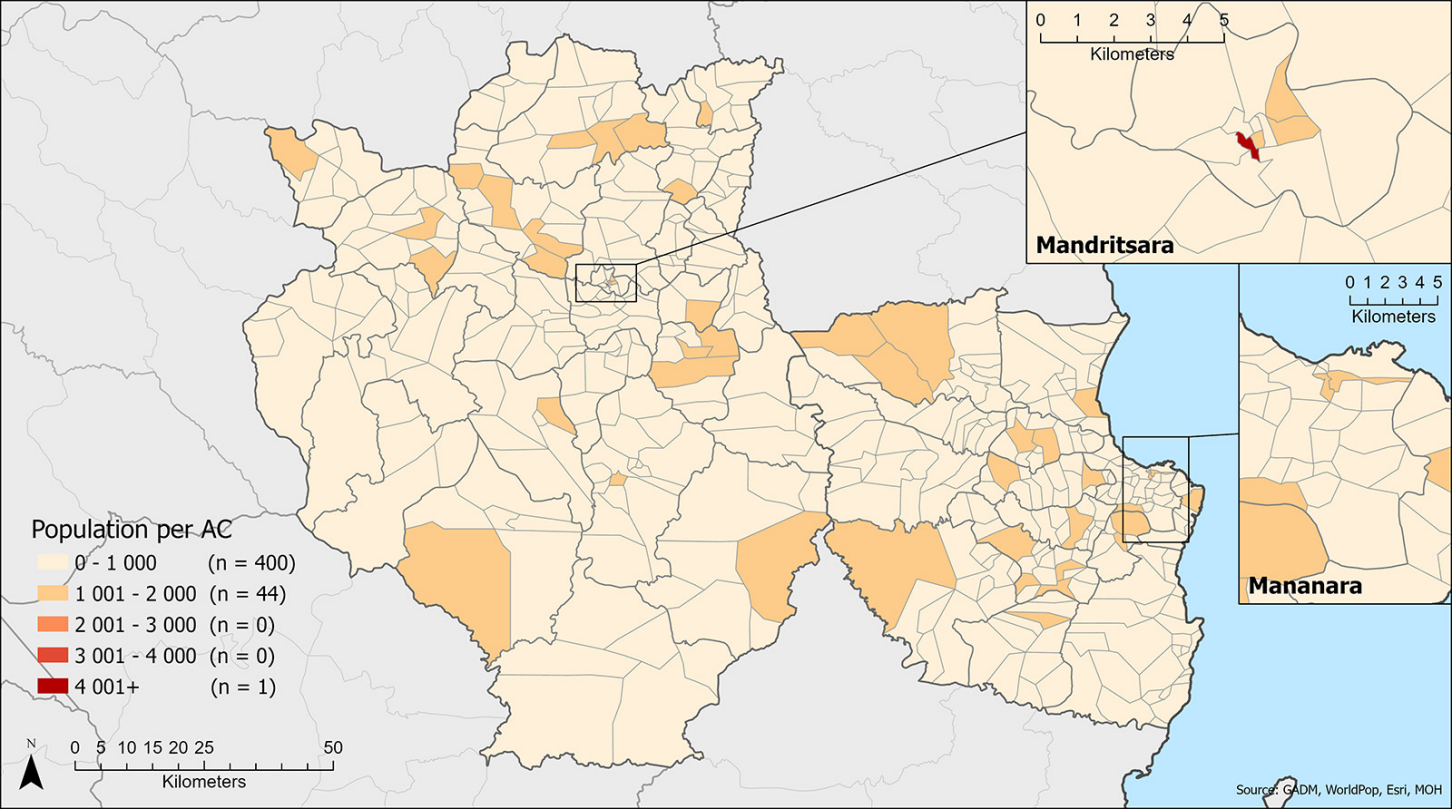
Population Coverage per ACs in Madagascar Abbreviation: ACs, agents communautaires (community health workers).

**FIGURE 3.**
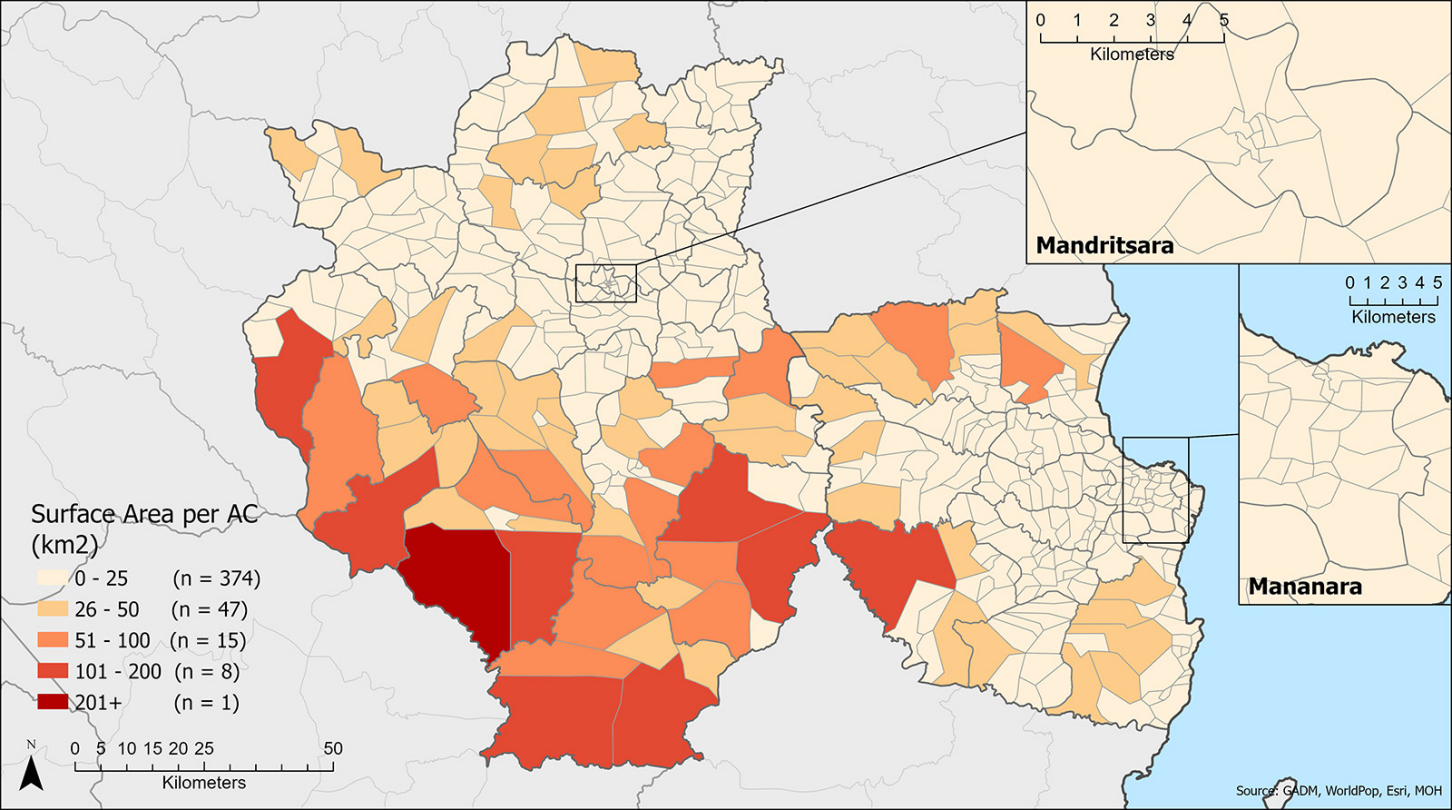
Surface Area to ACs in Madagascar Abbreviation: ACs, agents communautaires (community health workers).

Overall, 89% of fokontany had an estimated 2020 population of 2,000 or fewer people (range: 145–11,359) and 95% of fokontany spanned 50 km^2^ or less, with the largest fokontany covering 365.4 km^2^ (range: 0.1–365.4 km^2^). The population-to-AC ratio was at or below 1,000 in 90% of fokontany, and all but one fokontany had an expected population coverage of 2,000 people or fewer per AC (Figure 2). The surface-area-to-AC ratio was 25 km^2^ or less in 84% of fokontany, with the expected surface area coverage exceeding 50 km^2^ per AC in 5% of fokontany (Figure 3). One AC had an assigned area of 363 km^2^.

**FIGURE 4.**
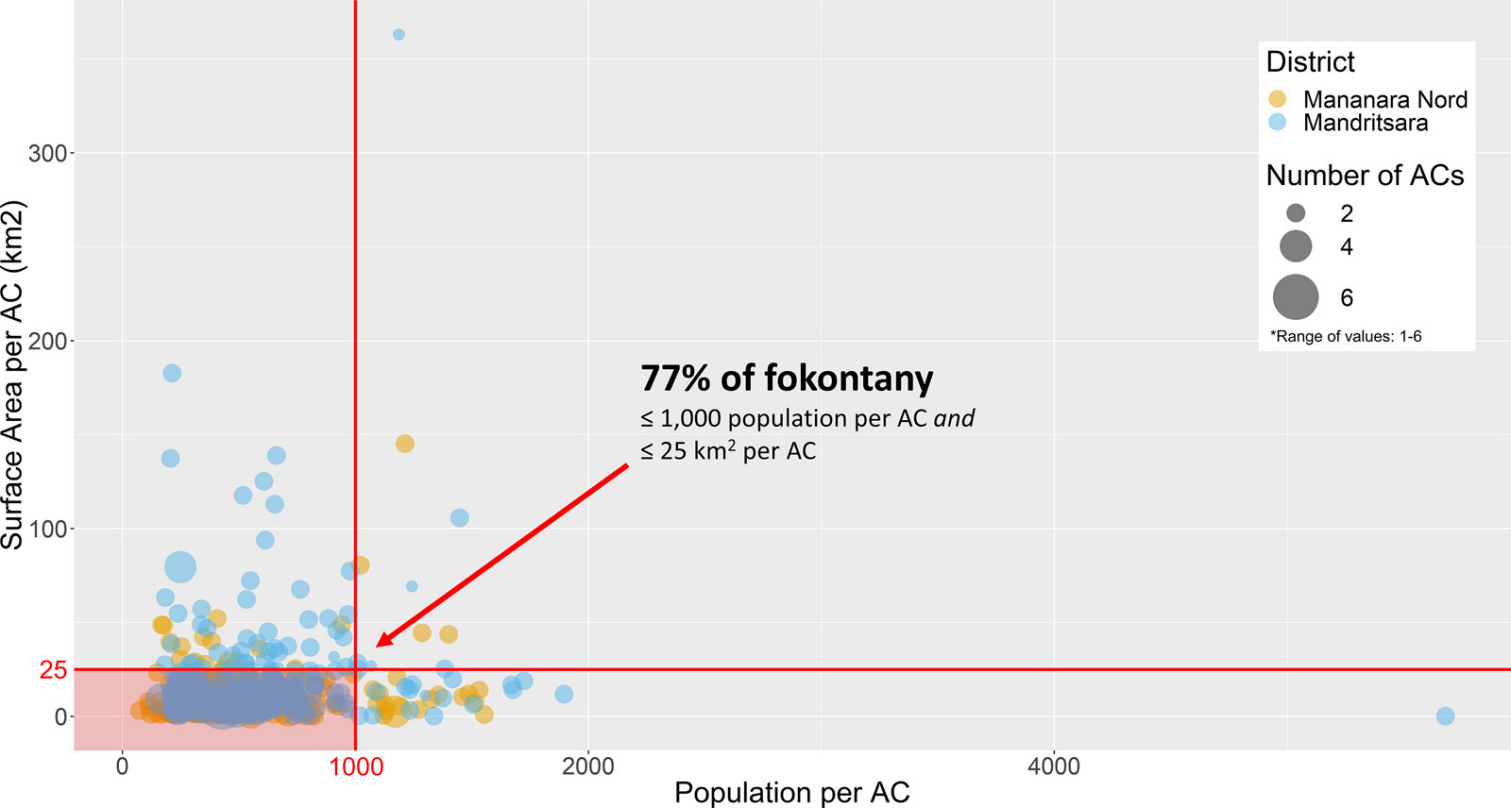
Number of Fokontany per Population and Surface Area Covered by ACs Abbreviation: ACs, agents communautaires (community health workers).

Figure 4 presents the overlay of population and surface area coverage. When combining the 2 measures, we found that 77% of fokontany in which ACs were assigned had 1,000 people or fewer and an area of 25 km^2^ or less.

### Travel Time

**FIGURE 5.**
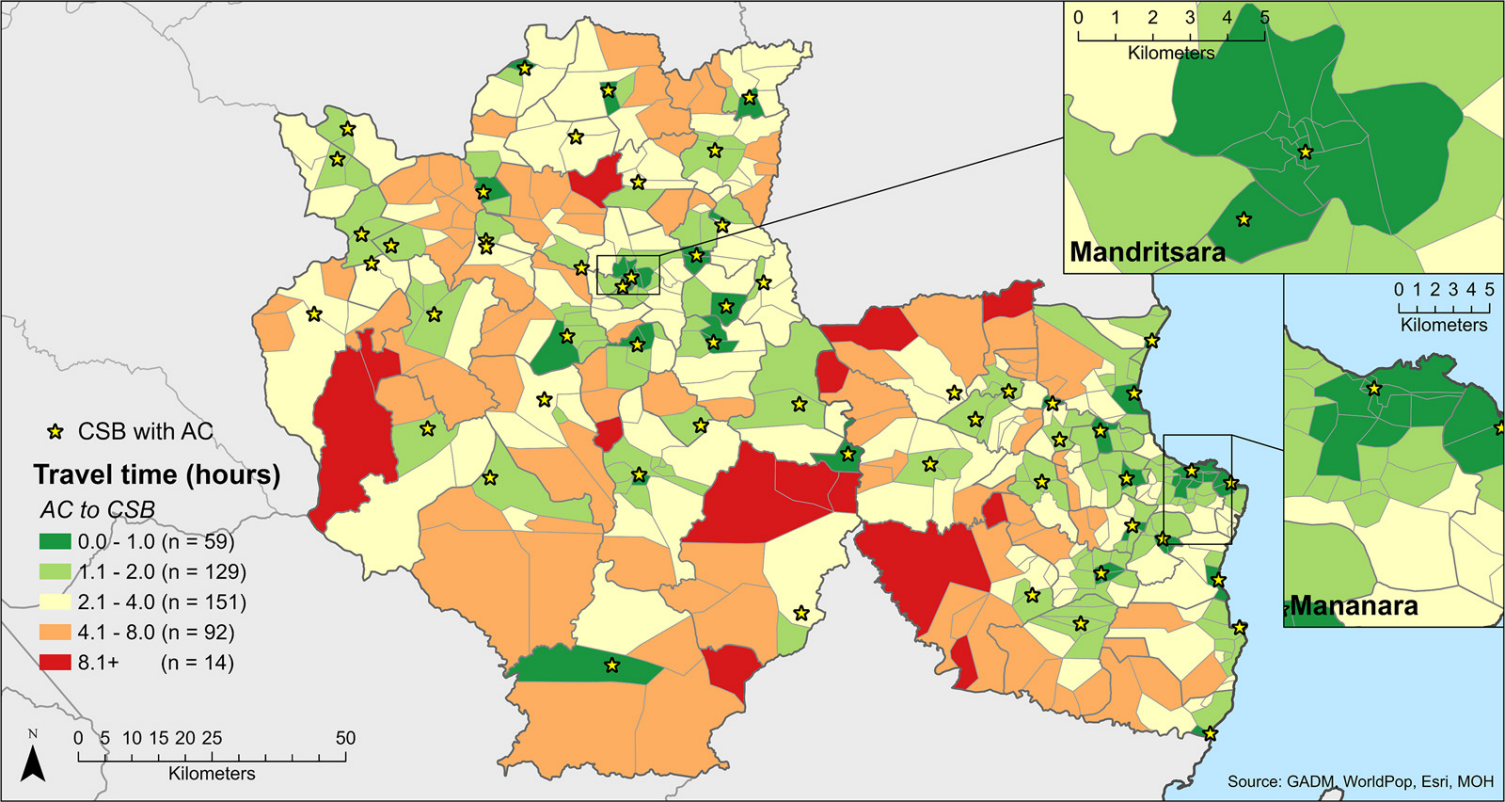
Estimated Travel Time for AC From Each Fokontany to Their Assigned CSB by Foot During the Dry Season (1 Way) Abbreviations: ACs, agents communautaires (community health workers); CSB, centres de santé de base (health centers).

**FIGURE 6.**
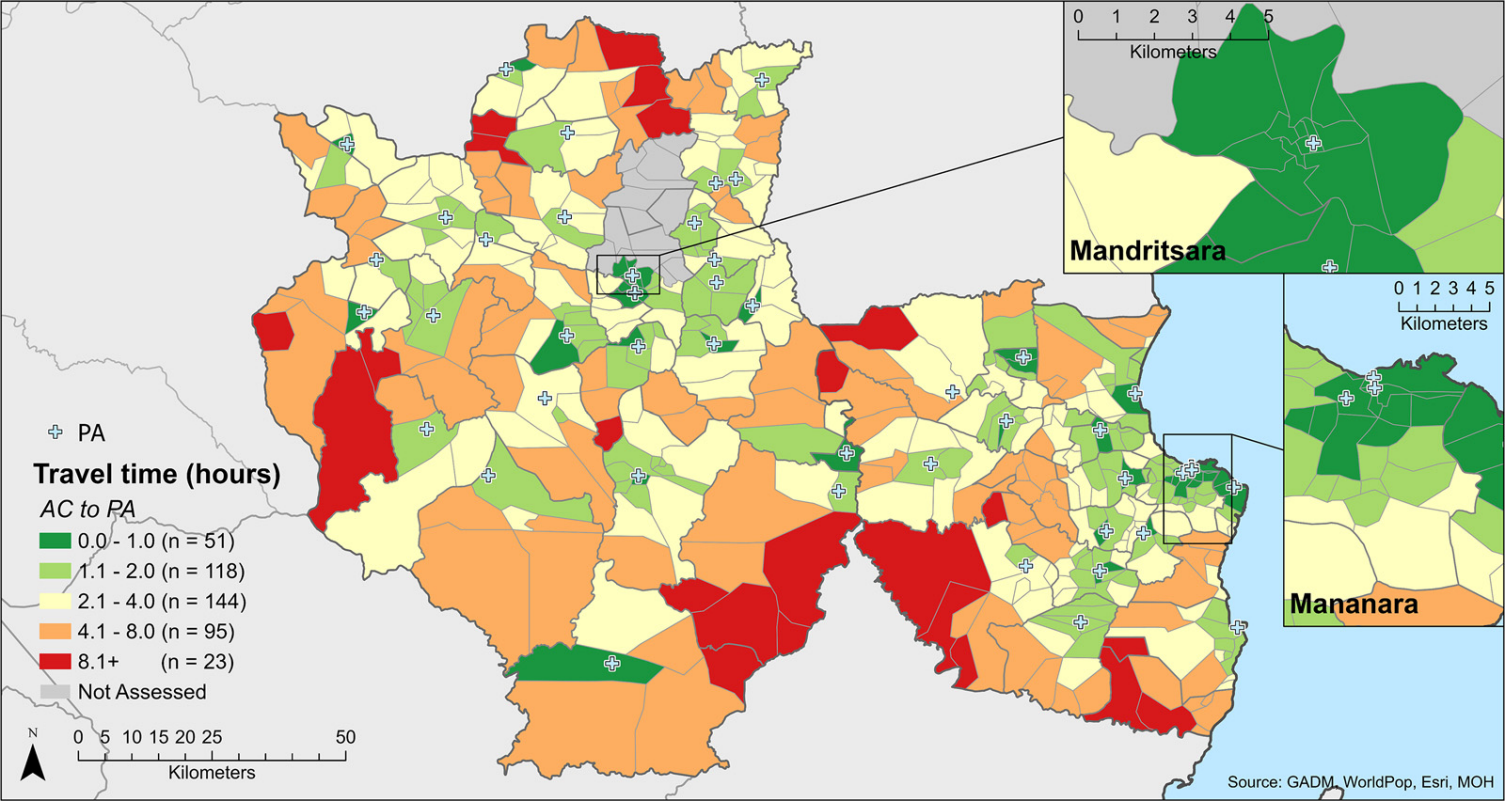
Estimated Travel Time for AC From Each Fokontany to Their Assigned PA by Foot During the Dry Season (1 Way) Abbreviations: ACs, agents communautaires (community health workers); PA, points d’approvisionnement (supply points).

Figure 5 and 6 show the estimated 1-way travel times to ACs’ assigned CSB and PA, respectively, by foot and under dry season conditions. ACs were within 2 hours by foot of their assigned CSB in 42% of fokontany, whereas travel time to the CSB was between 2 and 4 hours in 34% of fokontany and greater than 4 hours in 24% of fokontany. Travel time to the assigned PA was 2 hours or less in 39% of fokontany, between 2 and 4 hours in 33% of fokontany, and greater than 4 hours in the remaining 27%. The median travel time to the CSB and PA was similar (2.3 and 2.5 hours). In both cases, travel time varied greatly across fokontany, ranging from 3 minutes to more than 10.5 hours for CSBs and from 4 minutes to over 11 hours for PAs (Table 2).

**TABLE 2.** Travel Times for ACs From Each Fokontany to Their Assigned and Closest CSBs

**District**	**Assigned, h**				**Closest, h**			
	**Min**	**Max**	**Mean**	**Median**	**Min**	**Max**	**Mean**	**Median**
Travel time to CSB								
Mandritsara	0.05	10.59	3.00	2.56	0.05	7.62	2.52	2.20
Mananara Nord	0.13	9.17	2.68	2.17	0.13	8.41	2.24	1.89
Both districts	0.05	10.59	2.86	2.34	0.05	8.41	2.39	2.04
Travel time to PA								
Mandritsara	0.07	11.82	3.23	2.57	0.07	11.82	2.87	2.33
Mananara Nord	0.14	11.05	2.97	2.34	0.14	8.97	2.59	2.07
Both districts	0.07	11.82	3.11	2.45	0.07	11.82	2.74	2.27

Abbreviations: AC, agents communautaires (community health workers); CSB, centres de santé de base (health centers); PA, points d’approvisionnement (supply points).

Overall, ACs in 31% of fokontany were not assigned to their closest CSB, and ACs in 25% of fokontany were not assigned to their closest PA. After reassigning all ACs from their assigned to their closest CSB, the mean travel time decreased from 2.86 hours to 2.39 hours. The proportion of fokontany with ACs within 2 hours of travel time to the CSB increased from 42% to 49% and that of fokontany with travel time between 2 and 4 hours from 34% to 38%, and the proportion of fokontany where ACs were confronted with more than 4 hours or travel decreased from 24% to 13%.

Reassignment from the assigned to the closest PA led to a reduction in mean travel time from 3.11 hours to 2.74 hours. Similar to CSBs, there was an increase in the proportion of fokontany with a travel time of 2 hours or less (39% to 44%) or between 2 and 4 hours (33% to 35%) and a decrease in the proportion of fokontany with the least favorable travel time, over 4 hours (27% to 21%).

## DISCUSSION

We used GIS to support program managers in visualizing data patterns related to the current deployment patterns of ACs in 2 districts of Madagascar. Our focus was primarily on improving understanding of how policy guidelines translate to local realities in terms of the demands that are placed on ACs in the exercise of their tasks. This included describing the population and geographic coverage expected of ACs and the travel time demands associated with maintaining functional linkages to the health system. These analyses are not intended to identify specific benchmarks for a well-functioning program. Rather, they are meant to provide managers with information they can take into consideration to assess whether the status quo is rational and realistic and then to develop targeted plans for adaptive management.

These analyses are meant to provide managers with information for assessing whether the status quo is rational and realistic and developing plans for adaptive management.

Selection of ACs is a collaboration between community members, fokontany leaders, and the head of the CSB. Although CSB heads have discretion to add volunteers in situations in which it is warranted by specific local challenges, we found that the current deployment of ACs across the 445 fokontany in the 2 districts largely aligned with the guideline in use of 2 ACs per fokontany that is supported by implementing partners. With the current distribution, ACs in 90% of fokontany have a catchment population of 1,000 people or fewer (2020 estimates) and ACs in 84% of fokontany have a catchment area of 25 km^2^ or less. In 23% of fokontany, ACs are assigned either more than 1,000 people or more than 25 km^2^. These results only provide rough estimates given several assumptions made about the specific location of ACs and population patterns within fokontany. They should also be interpreted as upper limits since not everyone within the community may need or seek the services of ACs and ACs’ functions typically involve a combination of home visits and consultations at the health hut (toby). Import-antly, the thresholds we used (1,000 persons and 25 km^2^) are based on descriptive patterns observed in our data rather than an informed determination of what may be manageable. Experience from other countries and the WHO guideline on CHW programs indicate that there is no single, ideal target population size per CHW but rather that the optimal coverage ratio depends on a constellation of factors, including local epidemiology, CHWs’ scope of practice, geographic distribution of the population, geographic accessibility, and the balance between time demands and compensation and incentives.^6^^,^^11^ Thus, policy makers need to take these other factors into consideration alongside our findings and allow greater latitude for local communities and CSBs to reasonably select and manage the number of volunteers based on their circumstances. Our results offer an important first step in contextualizing guidelines and assessing whether resulting demands on ACs are inherently realistic.

Our results offer an important first step in contextualizing guidelines and assessing whether resulting demands on ACs are inherently realistic.

Overall, our analyses show that a set number of ACs per fokontany can mask some heterogeneity—and in some cases large differences—in population-to-AC and/or surface-to-AC ratios within and across districts. Similar coverage ratios may mask further differences because these figures do not capture possible variations in population distributions or terrain across fokontany. Although mainly discussed in the context of incentives, fairness and equitability have been noted as important to minimize frustration and attrition.^33^^–^^35^ Thus, the extent to which assignments per fokontany may result in variable expectations warrants additional attention. Madagascar may benefit from flexible guidelines calling for adjustments to recommended coverage ratios based on relevant contextual variables, as is currently the practice in some other countries based on distance from health facility (Liberia), urban versus rural settings (India and Zambia), or terrain (India and Nepal)^11^.

In modeling 1-way travel times by foot under dry season conditions, we estimated that ACs in 58% of fokontany were located more than 2 hours from their supporting health facility; for supply points, this proportion was 61%. Assignment to health facilities and supply points is currently governed by 2 different sets of boundaries—the “sectorization” or catchment areas served by health facilities and administrative boundaries, respectively. In practice, the duality of the health facility/supply point support system combined with inconsistent assignment rules can lead to irra-tional and suboptimal outcomes in terms of time demands on ACs. Our models reassigning ACs to the closest health facility did not make a substantive change in alleviating the travel time demands on ACs. We found only modest improvements that still left ACs in over half of fokontany with travel times exceeding 2 hours. Once adding time for meetings and a return trip, many ACs may effectively be required to forego a full day or leave their community overnight each time they travel to the health facility or supply point.

Under the Mahefa Miaraka program, ACs are allowed to sell at a small markup to the actual cost of health commodities and they also receive a modest allowance and a travel reimbursement (based on MSANP guidelines) for attending meetings that may not be sufficient to offset opportunity costs and possible expenditures, particularly for ACs who travel the farthest distances. Further-more, with a Rural Access Index of 11.4%, an estimated 17 million people in rural areas of Madagascar are unconnected to the road network (i.e., do not live within 2 km of the nearest road in good condition).^36^ Thus, the link between ACs and the health system warrants significant attention and may require different solutions beyond the reassignment explored in our analyses.

Although endowing ACs with bicycles, as has been done in some contexts,^6^ may cut travel times, this solution is not currently considered under the National Strategic Plan for Strengthening Community Health, largely because ACs are supported solely through partner-supported programs. In addition, bicycles would be difficult to use in the program area because of the sandy, muddy terrain during the rainy season. One option that is currently being discussed by the MSANP and implementing partners is to switch to a single-source resupply model exclusively through health facilities, suppressing the need for additional trips to supply points. Complementary measures ensuring that a monthly trip to the health center can serve as a 1-stop shop for supervision, reporting, and resupply to minimize travel burden should be considered. These may include commodity security at the health facility level, strong commodity management skills at the AC level, reliable communication systems to avoid unnecessary trips when supplies are lacking or staff is not available, and AC incentives. Transport and logistics challenges are compounded during the rainy season when some areas are entirely cut off, largely due to swelling of rivers. Rainfall patterns vary across Madagascar and have been disrupted due to changes in climate, with northern Madagascar receiving 150% of expected annual rainfall in 2018.^37^ Adaptive management that considers the dynamic impact of the rainy season on access will be increasingly important to reduce service disruptions and increase health system resilience. Additional creative interventions may be required for reliable access to communities, such as using hovercrafts for deliveries as was recently tested in another program.^38^

### Limitations

Analyses were conducted in 2 districts of Madagascar supported by the same community health program and may not adequately represent other areas of the country. As in any modeling exercise, our results are anchored in a combination of hard data and assumptions, for example, about the precise location of ACs within each fokontany. Obtaining geocoordinates for each AC was cost prohibitive and not feasible within the timeline of the project. Although our modeling assumptions were grounded in discussions with local staff, findings should be interpreted cautiously as directional rather than as a precise representation of the actual conditions that exist in the 2 districts. Population and geographic coverage are unlikely to be equally divided between ACs within the same fokontany. One of the most critical assumptions related to estimation of delays with river crossings is limited by the availability of data on river characteristics. In addition, modeling was performed under dry season conditions; a similar approach could be used with rainy season parameters and would likely lead to increased travel times. When interpreting findings, program managers should also consider how differences in the profile of ACs, such as those based on gender, age, occupation, or family responsibilities may affect reasonable expectations in terms of the demands placed on ACs.

## CONCLUSION

A major contribution of CHW programs is to bridge the gap between communities and formal health services. An earlier study in another area of Madagascar showed that CHWs share in the living conditions of the populations they serve.^39^ Complementing these findings, our analyses highlight that, although CHW programs reduce challenges for the client population to access an integrated package of services for women and children, access barriers are in fact transferred onto CHWs.

Using GIS to visualize the deployment patterns of CHWs can improve program managers’ ability to synthesize information and grasp the actual implications of policy decisions, and modeling within a GIS enables identification of data patterns related to the demands placed on CHWs, providing useful information to inform decision making. This information is timely to inform the MSANP’s strategic thinking around criteria and processes for optimal integration of ACs into communities and the health system in the context of the development of the National Strategic Plan on Strengthening Community Health. Our findings suggest that policy makers should consider allowing greater latitude to reasonably select and manage the number of ACs that match the realities of each community. In addition, program managers should consider assignment of ACs and placement of supply facilities that decrease travel time.

More broadly, optimization will require additional research to more fully understand the realities of ACs, and management and real-time data use to assess what is required to ensure functional support systems. Specific recommendations to balance these considerations include the following:

1. Conduct additional geospatial analysis including a larger sample of geographic areas to obtain a broader representation of the local realities of ACs in the country. Incorporate additional analysis of existing data from the HMIS to examine how coverage and travel time may correlate with functioning of community health systems.
2. Use task analysis and time-use research to better understand how ACs manage their tasks and whether expectations and workloads are realistic and commensurate with compensation and incentives.
3. Use costing tools to provide an accompanying estimate of the real costs of maintaining a volunteer community health system and required inputs.
4. Review management data on health staffing at CSBs to assess the ability of the health system to effectively work with groups of ACs, including for supervision and to ensure the quality of reporting, review, restock of supplies and continued learnings during monthly meetings.

## Notes

**Peer Reviewed**

**First published online:** August 28, 2020
